# Trends and Practices on Blood Flow Restriction Training Are Not Largely Aligned With the Contemporary Evidence

**DOI:** 10.7759/cureus.81766

**Published:** 2025-04-05

**Authors:** Vasileios Korakakis, Alexandra Korakaki, Themida Korakaki, Stefanos Karanasios, Roula Kotsifaki

**Affiliations:** 1 Department of Health Sciences, University of Nicosia, Nicosia, CYP; 2 Department of Physical Education and Sport Science, University of Thessaly, Trikala, GRC; 3 Physiotherapy, Hellenic Orthopaedic Manipulative Therapy Education, Athens, GRC; 4 European School of Physiotherapy, Amsterdam University of Applied Sciences, Amsterdam, NLD; 5 Department of Cultural Anthropology and Developmental Sociology, Vrije University, Amsterdam, NLD; 6 Physiotherapy, University of West Attica, Athens, GRC; 7 Department of Rehabilitation, Aspetar Orthopaedic and Sports Medicine Hospital, Doha, QAT

**Keywords:** bfr training, blood flow restriction, clinical practice, rehabilitation, safety

## Abstract

Objective: To evaluate trends and current clinical practice of physiotherapists on blood flow restriction training (BFRT) application.

Methods: An online survey was conducted to assess: a) demographics and professional characteristics, b) specifics of BFRT application, and c) safety and adverse events. We tested using Pearson’s Chi-square test whether the physiotherapist's characteristics were independent of their years of experience and formal BFR education.

Results: Most respondents reported having much confidence (n=47, 44.6%) in using BFRT, and they used it for a mean of 2.6±1.7 years. Significant variability among respondents was found in devices used, limb occlusion pressure calculation methods, the reperfusion scheme, the number of exercises implemented, and the percentage of complete occlusion pressure used for exercising. Most used BFRT in musculoskeletal conditions of the upper and lower limb (n=88, 86.3%), aiming improvements in strength and muscle volume (n=93, 90.3%), by using external load (n=82, 79.6%). The majority of the respondents (n=69, 67.0% attended a short course for BFRT, of which 55.1% (n=56) believed it was not evidence-based. No significant associations were found between the years of experience or attendance in a BFRT course with practices and perceptions of the surveyed physiotherapists (all p>0.05).

Conclusion: Current BFRT practices are largely not aligned with contemporary scientific evidence and recommendations.

## Introduction

In the last few years, the spotlight has increasingly focused on low-load blood flow restriction training (BFRT), a relatively novel intervention that combines low-load resistance with partial BFRT by applying inflatable cuffs at the most proximal part of the exercised limb. This technique has attracted attention for its potential benefits across various populations and conditions. Research indicates that low-load BFRT can induce muscle hypertrophy and increase strength in the healthy population, distal and proximal to its application [1,2]. Moreover, it shows promising results in attenuating muscle disuse atrophy in load compromised individuals [3], and improving muscle strength in individuals suffering weakness due to various musculoskeletal conditions [4-6]. In addition, contemporary evidence suggests that BFRT can decrease anterior knee pain [7,8] and improve pain and disability in patients with upper limb pathologies, such as lateral elbow tendinopathy [9].

However, while the benefits are evident, it’s crucial to note that reckless application of BFRT can lead to serious adverse effects [10] including pulmonary embolism, deep vein thrombosis, and rhabdomyolysis [11]. Nevertheless, literature on BFRT has provided guidelines for safe implementation and risk stratification [12], with studies suggesting that the adverse events are comparable to those seen in traditional strength training [11].

Though the efficacy and safety of BFRT have been widely studied and documented, the application of the intervention in the clinical setting is not yet fully assessed, appraised, and elucidated. Reports on BFRT by physiotherapists and strength and conditioning trainers in healthy and clinical populations reveal varying practices, with some aligning closely with contemporary evidence while others diverge [13]. Discrepancies in findings among studies may be due to the different publication dates of the reports, and the evolution of the trending practices in BFRT such as methods of limb occlusive pressure (LOP) calculation, the increased availability of auto-inflated or autoregulated devices, and the significant increase of the BFRT publications the last few years. Therefore, the main objectives of this study were to investigate the trends of BFRT in the clinical practice of Greek physiotherapists, to evaluate whether the application follows evidence and recommendations, and to explore safety topics.

## Materials and methods

Study design

We utilized an open online survey to conduct this study. Ethical approval was granted by the University of Thessaly Ethics Committee (ID: 4-2/09-12-2020), and a convenience sample of physiotherapists was recruited, and they gave informed consent. All data were collected through an online survey platform (SurveyMonkey, www.surveymonkey.com). Responses were stored securely in an encrypted database accessible only to the principal investigator and authorized team members. Any identifiable information collected was anonymized before analysis, and all personal data were securely deleted from the platform following the completion of the study. The study adhered to relevant guidelines, ensuring that no personal data was retained beyond the necessary period for analysis. The survey was promoted on mainstream social media, physiotherapy groups, and through the Panhellenic Physiotherapy Association network between June 2022 and November 2023, while no incentives were offered.

Survey design

Two physiotherapists experienced in survey design and applying BFRT in clinical rehabilitation generated the initial items. Three different item sources were utilized: literature review, clinical expertise, and identified relevant items from previously published BFRT surveys [13,14]. At the second stage, four physiotherapists independently (clinicians and academics) screened the item pool based on content, and in a consensus meeting, edited and removed duplicate, redundant, or irrelevant items. The initial 47 items were tested through a structured content analytic method [15]. The potential items were distributed to five judges (academics and physiotherapists) that matched each of the 47 items, based on their content relevance to the survey objectives, to a five-point Likert scale (1=poor to 5=excellent relevance) allowing judgments to be tested through quantitative statistical procedures using Aiken’s V statistic [16]. The V values are compared against a right-tailed binomial probability table, while values range from 0 to 1 (1=perfect agreement). From the items evaluated, 15 were excluded with V<0.70, thus 33 items with V values ranging from 0.75 to 1.0 (p<0.01) were retained for pilot testing.

The survey consisted of three sections: a) demographics and professional characteristics (items 1-8), b) the specifics of the BFRT application (items 9-34), and c) risk stratification, adverse events, and safety considerations (items 35-41). The 41-item anonymous survey is presented in Table 1. The responses in items with multiple selections/choices were randomized to prevent biases.

**Table 1:** The items of the anonymous blood flow restriction survey and counts of answered items from the included sample (n=103)

Item	Questions	Counts
Item 1	What is your sex?	103
Item 2	Please write your age in years	103
Item 3	What is the highest academic qualification you hold as a physical therapist?	103
Item 4	Do you have any clinical or professional specialization or certificate expertise through seminars or educational courses?	103
Item 5	How many years of work experience do you have?	103
Item 6	What best describes your current working status?	103
Item 7	In which area/region do you practice?	103
Item 8	Approximately how many years have you been using Blood Flow Restriction in your clinical practice?	103
Item 9	How confident are you in implementing Blood Flow Restriction training?	103
Item 10	How did you learn about Blood Flow Restriction training?	103
Item 11	What equipment / device do you use for the Blood Flow Restriction training?	103
Item 12	Why do you use Blood Flow Restriction?	103
Item 13	On which part of the body do you use Blood Flow Restriction?	103
Item 14	How often (on average) do you apply Blood Flow Restriction to patients, clients, physically active individuals, or athletes?	103
Item 15	In combination with what type of exercise/activity you use Blood Flow Restriction?	103
Item 16	Have you had any training or formal education in the use of Blood Flow Restriction training?	103
Item 17	Was your participation in formal training/education in conjunction with promotion or co-sponsorship by a company/distributor of blood flow restriction devices?	103
Item 18	Do you believe that the training provided to you was evidence based with regards to the application of Blood Flow Restriction?	103
Item 19	Do you believe that training should be mandatory before the clinical application of Blood Flow Restriction to patients, clients, physically active individuals, or athletes?	103
Item 20	Do you have doubts, concerns, or difficulties regarding the use of Blood Flow Restriction in your clinical practice?	100
Item 21	What barriers, concerns, or difficulties do you have regarding the use of Blood Flow Restriction in your clinical practice?	60
Item 22	How many exercises do you usually prescribe in a session with Blood Flow Restriction in your clinical practice?	103
Item 23	How long does a Blood Flow Restriction session usually last in total (including rest)?	103
Item 24	How long does the blood flow to the exercising limb remain restricted without reperfusion with the application of Blood Flow Restriction?	103
Item 25	How do you calculate the percentage or occlusion pressure in the exercising limb in a session with Blood Flow Restriction?	102
Item 26	In what position are you placing the patient/individual to determine the complete limb occlusion pressure and subsequently the percentage of occlusion of the exercising limb in a Blood Flow Restriction session?	102
Item 27	Do you re-calculate the complete occlusion pressure on the exercising limb each time you prescribe a different exercise (in the same session)?	102
Item 28	Do you re-calculate the complete occlusion pressure on the exercising limb in each training session (on different days)?	102
Item 29	What percentage or millimeters of mercury (mmHg) do you usually use for upper extremity blood flow restriction?	103
Item 30	What percentage or millimeters of mercury (mmHg) do you usually use for lower extremity restriction?	103
Item 31	How do you determine the external load or resistance in a Blood Flow Restriction session?	103
Item 32	How many sets and repetitions per set do you prescribe per exercise in a Blow Flow Restriction session?	103
Item 33	How much resting time do you give between sets of each exercise in a Blood Flow Restriction session?	102
Item 34	In which of the following conditions would you perform an intervention with Blood Flow Restriction?	103
Item 35	Have you performed sessions with Blood Flow Restriction for people with comorbidities, such as diabetes, hypertension, obesity, etc. in your clinical practice?	100
Item 36	Have you performed sessions with Blood Flow Restriction for older or elderly people (>60 years) in your clinical practice?	100
Item 37	Have you observed adverse reactions or events during or after the Blood Flow Restriction session in your clinical practice?	97
Item 38	If you answered yes to the previous question – have you observed adverse reactions or events – Please choose which of the following have you observed?	39
Item 39	Do you believe that individualized restriction pressure in the exercising limb would reduce the likelihood of adverse reactions or events?	100
Item 40	Do you carry out a risk factor assessment / screening before a Blood Flow Restriction intervention?	99
Item 41	Which of the following do you consider as absolute risk factors for an intervention with Blood Flow Restriction?	99

Ten physiotherapists (five males and five females; age range 26-48 years) piloted the survey for feasibility (comprehensibility, ease of administration, length, and completion time), readability, and face validity. All raters gave a positive rating for all domains, except the survey length (25 minutes completion time - would affect the response rate). Finally, testing identified a few required formatting changes, typos, and adjustments to enhance readability.

Data analysis

Survey data was analyzed anonymously using SPSS software, version 21.0 (IBM Corp., Armonk, NY), JMP software, version 16.0 (JMP Statistical Discovery LLC, Cary, NC), and Microsoft Excel (Microsoft Corporation, Redmond, WA). All responses were included in the analyses regardless of missing data. Categorical data were expressed as counts and percentages, and continuous data as means and standard deviations (SD).

To test the hypothesis that the relative proportions of selected clinical practices, perceptions, knowledge, and strategies were independent of respondents’ years of experience (≤5 years or >5 years) and formal BFRT education through a short course [17], the Pearson’s Chi-square test of independence was used. In contingency tables with multinomial dependent variables, when the Chi-square test assumptions were not met, we collapsed categories accordingly and re-ran the procedure.

The level of significance was set at 0.05, but for multiple comparisons (R x 2 tables), Bonferroni adjustments were performed using the adjusted residuals. We reported Phi (φ) and calculated the odds ratio (OR), the 95% confidence intervals (95% CI), and respective p-values for dichotomous variables, and Cramer’s V for multinomial variables to quantify the strength of association.

## Results

Survey response

Two hundred and forty-eight (248) physiotherapists accessed the online survey, of which 145 were excluded because they did not answer any questions (41% response rate). The average completion time was 29 minutes, and the average number of days since the survey went live was 131 (SD=157 days). We did not conduct a power analysis to predefine the required sample size, given that BFRT is a relatively new method and hasn’t been included in the physiotherapy scope of practice.

Respondent characteristics

All Greek geographical locations were represented (Table 2). The mean age of the respondents was 34 years (range 24-61), most were male (n=77, 74.7%), working in private practice (n=87, 84.5%), and had a bachelor’s or a master’s degree in physiotherapy plus other clinical specializations (n=44, 42.7%; n=41, 39.8%). Their mean clinical experience was 10.2±6.6 years, while they were using BFRT in practice for a mean of 2.6±1.7 years (range six months to 10 years). Most respondents reported having much confidence (n=46, 44.6%), compared to those with very little confidence (n=32, 31.1%) or very high confidence (n=25, 24.3%) in the clinical use of BFRT.

**Table 2:** Demographic and descriptive characteristics of the study respondents (n=103) Values are presented as counts and percentages (%), unless otherwise indicated. †Other diplomas and certificates included: manual therapy, McKenzie method, acupuncture, clinical Pilates, soft tissue massage or mobilization techniques, FIFA medical diploma, sports specialization courses, dry needling. SD, Standard deviation; NA, Not applicable; BSc, Bachelor of Science; MSc, Master of Science; PhD, Doctor of Philosophy, BFR, Blood flow restriction.

Characteristic	%	(n)
Sex		
Male	74.7	(77)
Female	24.3	(25)
Prefer not to say	0.97	(1)
Age (mean±SD)	34.1	±7.7
Male	35.0	±8.4
Female	31.5	±4.5
Prefer not to say	35.0	NA
Primary practice setting		
Private practice	84.5	(87)
Academic institution	1.9	(2)
Sports team or federation	10.7	(11)
Home visits	2.9	(3)
Region of practice		
Attica	46.6	(48)
Central Greece (except Attica)	1.9	(2)
Macedonia	18.4	(19)
Thrace	2.9	(3)
Epirus	0.9	(1)
Thessaly	3.9	(4)
Peloponnese	6.8	(7)
Crete and islands	5.8	(6)
Cyprus	9.7	(10)
Other country	2.9	(3)
Qualifications		
BSc (only)	11.6	(12)
MSc	3.9	(4)
PhD plus other diplomas & certificates^†^	1.9	(2)
BSc plus other diplomas & certificates^†^	42.7	(44)
MSc plus other diplomas & certificates^†^	39.8	(41)
Time in practice as a physical therapist (mean±SD)	10.2	±6.6
BFR training experience (years)	2.6	±1.7
Confidence in using BFR in clinical practice		
No confidence at all	0.0	(0)
Very little confidence	31.1	(32)
Much confidence	44.6	(46)
Very much confidence	24.3	(25)

How physiotherapists learn about BFRT

**Figure 1:**
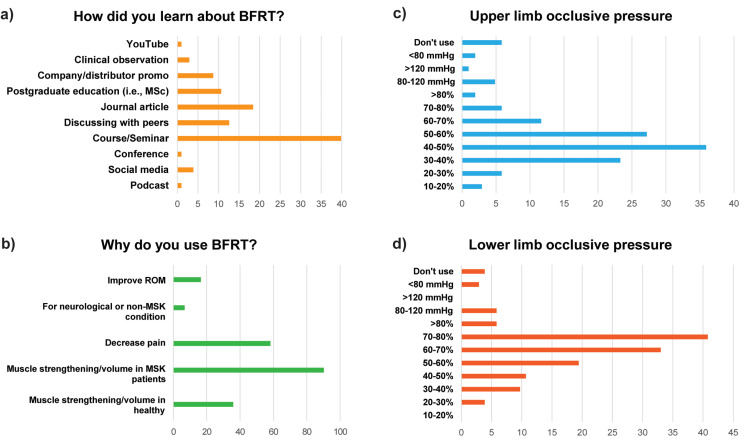
Physiotherapists responses in survey items enquiring about the application of BFRT in clinical practice Responses to questions: a) “How did you learn about Blood Flow Restriction training?”, b) “Why do you use Blood Flow Restriction?”, c) “What percentage or millimeters of mercury (mmHg) do you usually use for upper extremity blood flow restriction?”, and d) “What percentage or centimeters of mercury (mmHg) do you usually use for lower extremity restriction?” Note: Bars indicate percentages (%) of the total sample size (n=103). BFRT, blood flow restriction training; MSc, master’s degree; ROM, range of motion; MSK, musculoskeletal.

The responses indicated that BFRT entered physiotherapy practice by “attending private education short courses” (n=41, 39.8%), “scientific publications” (n=18, 18.4%), and “‘interactions with colleagues” (n=13, 12.6%), which comprised about 71% (n=73) of all responses (Figure 1a).

Clinical application of BFRT

Most of the surveyed physiotherapists used BFRT aiming to improve muscle strength and/or volume in patients with musculoskeletal conditions (Figure 1b). Most of the respondents used manual (n=42, 40.8%) or automatic (n=47, 45.6%) inflated cuffs. The clinicians determined LOP: automatically by the device (n=41, 39.8%), doppler ultrasound (n=34, 33.0%), perceived tightness scale 0-10 (n=10, 9.7%), limb circumference (n=7, 6.8%), standard mmHg values (n=6, 5.8%), pulse oximeter (n=3, 2.9%), and stethoscope (n=2, 2.0%). LOP was calculated with the patient in an exercise-dependent position (n=52, 50.5%), supine (n=33, 32.0%), standing (n=11, 10.7%), seated (n=6, 5.8%), and in prone position (n=1, 0.97%). For exercises within the session, 60.2% (n=62) do not recalculate LOP, while 64.1% (n=66) reevaluate the complete LOP every subsequent session. Patients receive BFRT on both upper and lower extremities by most of the respondents (n=89, 86.4%), with 12.6% (n=13) of the respondents using only on the lower extremity and 0.97% (n=1) only on the upper extremity. BFRT was administered by physiotherapists at varying frequencies: one to two sessions per week (n=47, 45.6%), two to three sessions per week (n=34, 33.0%), once every two weeks (n=15, 14.6%), or daily (n=7, 6.8%). The selection of the percentage of complete LOP used in clinical practice is illustrated in Figures 1c, 1d.

BFRT and exercise parameters

**Figure 2:**
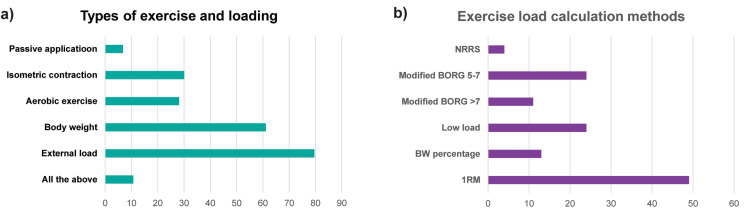
Physiotherapists’ responses in survey items enquiring about exercise loading during the application of BFRT in clinical practice. Physiotherapists’ responses in survey items enquiring about exercise loading during the application of BFRT in clinical practice. Responses to questions: a) “In combination with what type of exercise/activity do you use blood flow restriction?” and b) “How do you determine the external load or resistance in a blood flow restriction session?”. Note: In part (a), bars indicate percentages (%) of the total sample size (n=103), but in part (b), bars represent counts (multiple selection items). BFRT, blood flow restriction training; NPRS, numeric pain rating scale; 1RM, one repetition maximum; BW, body weight.

Resistance exercises were used with BFRT by most of the respondents (n=82, 79.6%), while a range of methods was utilized to determine the load (Figures 2a, 2b). The exercises prescribed ranged from 1 to >4 (one=11.7%, 1-4=45.6%, >4=1.9%), but some utilized an individualized number (n=42, 40.8%). Considering the sets and repetitions, most (n=73, 70.9%) used the standard described protocol in BFRT research (30, 15, 15, 15 repetitions), while 18.4% (n=19) used strength and conditioning principles (i.e., 3-4 sets by 6-12 repetitions), and 10.7% (n=11) prescribed exercises (3-4 sets) to volitional fatigue. Rest time used was 30-60 seconds by most respondents (n=53, 51.5%), 15-30 or >60 seconds was reported by 25.2% (n=26) and 20.4% (n=21), respectively, while 2.90% (n=3) used a resting time of <15 seconds.

The total BFRT session time ranged from 4 to 60 minutes, with most reporting 15-30 minutes (n=64, 62.0%). Significant variability was reported regarding the reperfusion and the time of limb occlusion; reperfusion between sets and between exercises 26.2% (n=27), reperfusion between exercises only 23.3% (n=24), no reperfusion at all 21.4% (n=22), reperfusion between sets only 12.6% (n=13), occlusion throughout the session 9.7% (n=10), and use of device-programmed protocols 6.8% (n=7).

BFRT and formal education short courses

**Figure 3:**
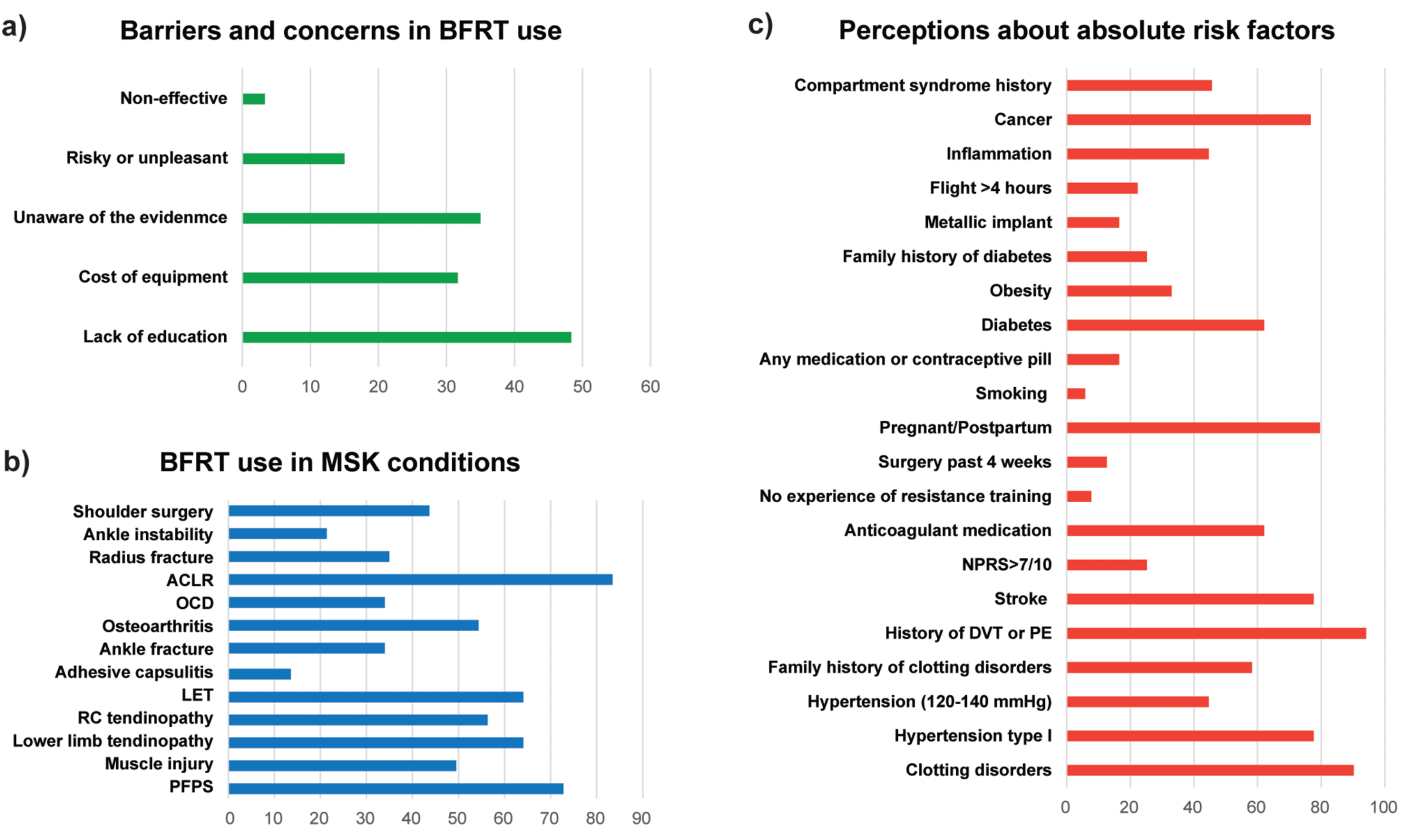
Physiotherapists’ responses in survey items enquiring about barriers and concerns in the application of BFRT, the absolute risk factors, and the use in musculoskeletal conditions. Responses to questions: a) “What barriers, concerns, or difficulties do you have regarding the use of blood flow restriction in your clinical practice?”, b) “In which of the following conditions would you perform an intervention with blood flow restriction?”, and c) “Which of the following do you consider as absolute risk factors for an intervention with blood flow restriction?”. Note: In parts (b) and (c), bars indicate percentages (%) of the total sample size (n=103), but in (a), bars represent the percentage of the respondents who identified barriers and have concerns about BFRT application (n=60). BFRT, blood flow restriction training; MSK, musculoskeletal; ACLR, anterior cruciate ligament reconstruction; OCD, osteochondral defect; LET, lateral elbow tendinopathy; RC, rotator cuff; PFPS, patellofemoral pain syndrome; NPRS, numeric pain rating scale; DVT, deep venous thrombosis; PE, pulmonary embolism.

Of the total, 67% (n=69) of the respondents have attended a course, of which 44.9% (n=46) believed that the training provided was evidence-based, and 42.0% (n=43) attended a course organized by the BFR company/distributor. 80.6% (n=83) believed that training should be mandatory before the clinical application of BFRT (no opinion 12.6%, n=13; disagree 6.8%, n=7). Barriers and concerns were reported by 58.3% (n=60) of the respondents, with the most prevalent being the lack of comprehensive evidence-based educational courses (n=58, 48.5%) (Figure 3a).

Patients, risk factor screening, and adverse events

All the respondents mostly use BFRT for musculoskeletal conditions (Figure 3b), while 32% (n=33) have used it in patients of >60 years of age, and 12.6% (n=13) in patients with a relatively higher risk due to comorbidities. Notably, 96% (n=99) reported performing pre-application screening (Figure 3c). The majority (n=89, 86.4%) reported that the individualized LOP calculation would significantly decrease the possibility of adverse events.

Adverse events from BFRT were reported by 40.7% (n=42) of the clinicians with the most severe being rhabdomyolysis (n=3, 2.9%) and the most frequent being delayed onset muscle soreness (n=61, 59.2%), dizziness/fainting (n=44, 42.8%), bruising or subcutaneous hemorrhage (n=44, 38.1%), sweating (n=32, 30.9%), pain increase (n=22, 21.4%), palpitation (n=20, 19.5%) and all other <10% (cramping, numbness, nausea).

Associations with participant years of clinical experience

No significant associations were found between the years of experience of the physiotherapists and their confidence in using BFRT (X^2^_(2)_ = 0.776; p = 6748), method of LOP calculation (X^2^_(2)_ = 1.198; p = 0.5493), position of calculation (X^2^_(2) _= 1.930; p = 0.3811), or application in patients with comorbidities (i.e., diabetes, hypertension, obesity) (X^2^_(1)_ = 2.856; p = 0.091).

A significant association (X^2^_(1)_ = 8.031; p = 0.0046) was found with a small effect (φ=0.28) between the years of experience and the application of BFRT in patients >60 years of age. The odds (OR=0.18; 95%CI: 0.498 to 0.649) of physiotherapists not implementing BFRT in patients >60 years were about two times higher if they were less experienced.

Associations with participant formal BFRT education

No significant associations were found between formal BFRT education of the physiotherapists' and their method of LOP (X^2^_(2)_ = 2.145; p = 0.3421), position of calculation (X^2^_(2)_ = 1.474; p = 0.4787), application in patients with comorbidities (i.e., diabetes, hypertension, obesity) (X^2^_(1)_ = 2.090; p = 0.1483) or in patients >60 years (X^2^_(1)_ = 0.002; p = 0.9618). An association (X^2^_(2)_ = 6.831; p = 0.0329) was found with a small effect (V=0.18) between formal BFRT education and physiotherapists’ confidence in the clinical application of the intervention; however, this association was not significant after the Bonferroni correction (p>0.0083).

## Discussion

This study investigated the current clinical practice and perceptions of Greek physiotherapists regarding the application of BFRT. The main finding was that physiotherapists’ practices are not largely aligned with the contemporary evidence supporting and informing BFRT, but are relatively safe. Several misconceptions were identified about the application of the method, such as using elastic bands without measuring LOP, aiming to improve joint range of motion, and allowing reperfusion between sets of exercises. Additionally, there were concerns about the calculation of complete LOP as approximately 30% (n=31) of respondents are using unreliable methods, such as perceived tightness or limb circumference [18]. There are also concerns about the percentage of occlusion of the exercised limb with high LOPs being applied for muscle strengthening in both upper and lower limbs [19,20]. Furthermore, there are instances of BFRT application in conditions without scientific evidence, such as adhesive capsulitis and a lack of superiority evidence over traditional safer interventions, such as knee osteoarthritis management with traditional exercise and education [5].

Nevertheless, about 90% (n=93) of the surveyed physiotherapists reported carrying out a risk assessment and believe that the percentage of LOP should be individualized, a practice that aids in the control and mitigation of the risk. This was reflected in the reported non-significant adverse events and the overestimation of absolute risk factors for the application of BFRT [12].

BFRT practices are not largely aligned with the evidence, perhaps because the evidence is not clear, or well disseminated 

Evidence suggests that journal articles are not commonly used as the main sources of information for physiotherapists to change clinical practice, with only 10% relying on them. Instead, about 50% of physiotherapists cited sources such as ‘interactions with colleagues’ and ‘attending private education short courses’ [21]. Similarly, these two categories of responses were predominantly selected by 52.5% (n=54) of the respondents in the present survey. It has been argued that ‘as a way of disseminating clinical research and influencing clinical practice, scientific publications aren’t achieving their original aims’ [21]. In line with this was the lack of significant associations between the formal education of the respondents, their perceptions, and clinical practices. Notably, a recent review concluded that only few continuing education courses on physiotherapy interventions are based on evidence provided by clinical practice guidelines (34%) or systematic reviews (20%), while course descriptions often misrepresented or contradicted the current state of evidence, and courses used insufficient or improper citation of the current body of literature [22].

We respectfully posit that the evidence available to clinicians is often unclear and in some cases invalid. Even in well-researched areas such as anterior cruciate reconstruction rehabilitation, significant variability and diversity in the practices of physiotherapists for clinical decision-making have been found worldwide [17,23]. Additionally, there are no published universal clinical practice guidelines on the application of BFRT. To date, 21% of the published literature for BFRT is reviews or systematic reviews (searched on PubMed in March 2024). These reviews often recycle the same original research in different publications. To illustrate, several systematic reviews [5,24,25] evaluating the effectiveness of low-load BFRT in patients with knee osteoarthritis have been conducted using the same five randomized controlled trials. Markedly, all used different strategies to analyze and present the data, and some, by conducting meta-analyses, reported inconsistent results due to critical errors that may have inadvertently crept into the final analyses, which in turn have influenced the ultimate outcomes (effect sizes) as calculated and reported [5,24,26]. We believe that those skilled in evaluating the quality of research and evidence and who have no financial interest in the educational short courses, the journal editors, the reviewers, the academics, can help inform the clinical world by filtering and directing the evidence to the correct channels, away from self-promotion, misinterpretation of research findings, selective presentation of evidence, and poor disclosure of conflicts of interest [21,22]. 

BFRT application, recommendations, and the evidence base

Interestingly, 68.9% (n=71) of the respondents reported high and very high confidence in the application, despite their relatively short experience in the use of BFRT (mean 2.6 years) and the fact that one out of three respondents were self-taught. Most physiotherapists implemented BFRT in patients with musculoskeletal conditions to improve strength and/or muscle volume (n=93, 90.3%) and to decrease pain (n=60, 58%) by largely using external load. However, the percentage of complete LOP employed was relatively high. Of total, 78.7% (n=81) and 46.7% (n=48) of the physiotherapists used >60% and >70% of the complete lower LOP, respectively, while 46.7% (n=48) and 18.5% (n=19) used pressures >50% and >60% in the upper limb, respectively. These LOPs have been mostly associated with favorable results in terms of pain reduction (but not strength improvements) [27] due to the difficulty and the exertion [28] in both patients and healthy individuals [29]. In addition, despite the contemporary evidence that body position strongly influences the complete LOP, suggesting to be calculated in the exercising position [28], still 50% (n=51) of the surveyed physiotherapists haven’t aligned their practice with the evidence and 27.2% (n=28) of the respondents use unreliable and invalid methods of LOP determination. Aligned with scientific publications [1,27], but not supported by clinical reasoning [18], was the clinical use by 71% (n=73) of the respondents of the most referenced repetition scheme in BFRT research (30, 15, 15, 15 repetitions). The low number of respondents reporting serious side effects (i.e., rhabdomyolysis) denotes that physiotherapists consider safety when prescribing BFRT; however, standardization of the application is much needed for the LOP determination methods, individualized pressures in the exercising position with adequate reperfusion schemes, and clinical reasoning driven application of BFRT by following the proposed recommendations found in relevant scientific literature.

Limitations

We acknowledge that a selection bias that may underestimate the true variability of respondents in Greece could be present as a limitation, as only physiotherapists using BFRT and being active in social media completed the survey. The researcher's positionality and approach of sharing the survey within their online social circle of physiotherapists, which subsequently evolved into snowball sampling, introduces another potential bias. This method likely attracted respondents who share similar beliefs, experiences, and perspectives as the researcher, thereby limiting the diversity of viewpoints represented in the survey responses. Additionally, the survey's reliance on a specific online social circle may not capture a wide range of perspectives, thus impacting the generalizability of the findings to practitioners with possibly opposing opinions or practices. Furthermore, the sociocultural and educational background of the respondents could shape their perspectives and approaches to BFRT, potentially affecting the interpretation and applicability of the results on a broader scale. Finally, the lack of gender-based analysis and the survey length may be regarded as limitations, with the number of items potentially explaining the dismally low response rate. The number of items may have overwhelmed respondents, discouraging participation and potentially skewing the sample towards those with stronger motivations or interests in the topic.

## Conclusions

Physiotherapists’ practices demonstrated significant variability and are not largely aligned with the contemporary evidence supporting and informing BFRT, although they prioritize safety when prescribing the intervention. Educational BFRT short courses influence clinical practice, but it seems that little verification is performed regarding the actual content quality and whether it is based predominantly on scientific evidence. Future research should be directed towards identifying and understanding the barriers in implementing research findings into practice, improving ways of research dissemination, and the uptake of research into practice.
